# Spore-Forming Thermophilic Bacterium within Artificial Meteorite Survives Entry into the Earth's Atmosphere on FOTON-M4 Satellite Landing Module

**DOI:** 10.1371/journal.pone.0132611

**Published:** 2015-07-07

**Authors:** Alexander Slobodkin, Sergey Gavrilov, Victor Ionov, Vyacheslav Iliyin

**Affiliations:** 1 Winogradsky Institute of Microbiology, Russian Academy of Sciences, Prospect 60-letiya Oktyabrya 7/2, 117312 Moscow, Russia; 2 Russian Federation State Research Center Institute of Biomedical Problems, Russian Academy of Sciences, Khoroshevskoe Shosse 76 A, 123007 Moscow, Russia; University of Padova, Medical School, ITALY

## Abstract

One of the key conditions of the lithopanspermia hypothesis is that microorganisms situated within meteorites could survive hypervelocity entry from space through the Earth’s atmosphere. So far, all experimental proof of this possibility has been based on tests with sounding rockets which do not reach the transit velocities of natural meteorites. We explored the survival of the spore-forming thermophilic anaerobic bacterium, *Thermoanaerobacter siderophilus*, placed within 1.4-cm thick basalt discs fixed on the exterior of a space capsule (the METEORITE experiment on the FOTON-M4 satellite). After 45 days of orbital flight, the landing module of the space vehicle returned to Earth. The temperature during the atmospheric transit was high enough to melt the surface of basalt. *T*. *siderophilus* survived the entry; viable cells were recovered from 4 of 24 wells loaded with this microorganism. The identity of the strain was confirmed by 16S rRNA gene sequence and physiological tests. This is the first report on the survival of a lifeform within an artificial meteorite after entry from space orbit through Earth’s atmosphere at a velocity that closely approached the velocities of natural meteorites. The characteristics of the artificial meteorite and the living object applied in this study can serve as positive controls in further experiments on testing of different organisms and conditions of interplanetary transport.

## Introduction

The transport of microorganisms in space is an important issue from theoretical (origin of life on Earth) and practical (planetary protection) perspectives. One of the most feasible mechanisms of interplanetary transport is the natural transfer of organisms in rocks (lithopanspermia). Presumably, this should include three main stages: ejection of the rock from a planetary surface, transit in space, and atmospheric entry [1–5].

Thermophilic anaerobic microorganisms are promising candidates for testing of the lithopanspermia hypothesis. Thermophiles (optimal temperature of growth above 50°C) are adapted to living at elevated temperatures and have a higher chance of surviving exposure to high temperatures. The majority of species of thermophilic bacteria and archaea are anaerobic organisms and do not need oxygen for growth. Thermophilic prokaryotes inhabit various environments, including the terrestrial subsurface, where they can be found in underground water and rock formations at depths down to 3 km [6]. It can be assumed that thermophiles also dwell in the subsurface of other planets and in cases of impact phenomena could be ejected into space inside their rocky habitat. Geochemical estimates of the temperature of the early Earth (earlier than 3.5 billion years) suggest that it was a much hotter planet that it is today [7, 8]. The reduced atmosphere most likely consisted of nitrogen, carbon dioxide, methane, ammonia, and carbon monoxide and was devoid of significant amounts of oxygen [9]. Having arrived on the Earth, thermophilic anaerobes could immediately and quickly proliferate and successfully colonize the high-temperature anoxic environment prevailing at that time.

Although lithopanspermia remains a hypothesis, all of the separate phases of this possible process have been experimentally tested. The survival of bacterial and yeast spores has been demonstrated in explosive and ballistic experiments which simulate hypervelocity impact and launch [10–12]. Numerous experiments in low Earth orbit performed on various spaceflight facilities have shown that with minimal UV shielding, several types of organisms can survive exposure to full space parameters for at least several months (reviewed in [5]). The longest time of survival has been recorded for *Bacillus subtilis* spores, which survived nearly 6 years in outer space [13].

The main factor affecting life during atmospheric entry is the high temperature, which is dependent on the velocity of the meteoroid. The survival of living organisms after atmospheric entry has been tested in experiments with “artificial meteorites”–rock samples fixed on sounding rockets or the landing capsules of space vehicles. Sounding rockets did not reach the velocities attained by natural meteorites during atmospheric entry (>10 km/sec). It has been shown that after atmospheric entry at a speed of 1.2 km/sec and a maximum recorded temperature of 145°C, up to 4% of *Bacillus subtilis* spores survived being infused into the surface of the granite samples attached to the exterior of the sounding rocket [14]. Plasmid DNA applied directly to the external surface of a sounding rocket also retained its biological function after suborbital flight [15]. Simulations performed with the landing capsules of space vehicles appear more realistic due to the higher entry velocities; however all experiments with microorganisms, which so far have been conducted exclusively on FOTON-M missions, have produced negative results. In the STONE-5 experiment, spores of *B*. *subtilis* and the fungus *Ulocladium atrum* and vegetative cells of the cyanobacterium *Chroococcidiopsis* sp. were placed into different rock samples with a thickness of 1 cm, and the top surface of one rock was additionally soaked with *Chroococcidiopsis* sp. The samples were mounted on the heat shield of the FOTON-M2 capsule and underwent hypervelocity atmospheric entry (7.6 km/sec); however no viable microorganisms were recovered after the flight [16, 17]. In the STONE-6 experiment (FOTON-M3 mission, 7.6 km/sec), the cyanobacterium *Chroococcidiopsis* located on the back side of a 2-cm-thick chert sample and lichen *Rhizocarpon geographicum* protected by a thin cover of glass textolite also did not survive atmospheric entry [18, 19].

Bacterial endospores are the hardiest known forms of life [20]. Spores of thermophilic bacteria have the highest heat resistance: endospores of *Moorella thermoacetica* had a 111-min decimal reduction time (the time of exposure to reduce viable spore counts by 90%) at 121°C, while endospores of *Desulfotomaculum* spp. survived triple autoclaving and 15-min exposure to 140°C [21, 22]. Among the spore-forming thermophiles, *Thermoanaerobacter siderophilus* SR4^T^ has one of the highest temperatures for growth (optimum: 70°C; upper limit: 78°C); spores of this microorganism survive autoclaving at 121°C for 90 min [23]. *T*. *siderophilus* is an anaerobe capable of dissimilatory reduction of insoluble Fe(III) oxides, the physiological property that could enable its development in endolithic environments.

The results presented in this paper were obtained during the METEORITE experiment on board the FOTON-M4 satellite. The aim of the METEORITE experiment was to test the possibility of the survival of microorganisms within an artificial meteorite after hypervelocity entry from space through Earth’s atmosphere.

## Materials and Methods

### Bacterial strain and culture conditions

*Thermoanaerobacter siderophilus* SR4^T^ (= DSM 12299^T^) was obtained from the German Collection of Microorganisms and Cell Cultures (Leibniz Institute DSMZ). It was routinely cultured in the laboratory in a liquid anaerobic medium with peptone as an electron donor and ferrihydrite (poorly crystalline Fe(III) oxide) as an electron acceptor at 65°C. The cultivation medium was prepared anaerobically by boiling and cooling it under CO_2_ (100%) gas phase. The medium contained (per liter of distilled water): 0.33 g of KH_2_PO_4_, 0.33 g of NH_4_Cl, 0.33 g of KCl, 0.33 g of MgCl_2_.2H_2_O, 0.33 g of CaCl_2_.2H_2_O, 2.0 g of NaHCO_3_, 5.0 g of peptone (Fluka), 0.2 g of yeast extract (Difco), 1 ml of trace-element solution [24], and 10 ml of vitamin solution [25]. The pH was adjusted to 6.5–6.8 (at 25°C) with 10% (w/v) NaOH. No reducing agent was added to the medium. Fe(III) was provided in the form of ferrihydrite at 90 mmol Fe(III) per liter. Ferrihydrite was synthesized by titrating a solution of FeCl_3_.6H_2_O with 10% (w/v) NaOH to pH 9.0. The pH of the autoclaved medium measured at 65°C was 6.8–6.9. Cultures were grown in 10 ml of medium in 17-ml Hungate tubes under an atmosphere of CO_2_ (100%). All transfers and sampling of cultures were performed with syringes and needles. The medium was heat-sterilized at 135°C for 60 min. For preservation of the dried cultures, different iron-containing minerals were used as excipients (matrix-forming additives). Three excipients were applied: (i) a mixture of magnetite/siderite formed in the medium with ferrihydrite (90 mM of Fe(III) per liter) due to microbial Fe(III) reduction in the course of growth [23]; (ii) glauconite (structure confirmed by X-ray diffraction analysis), added to the medium instead of ferrihydrite (100 mg in 10 ml); and (iii) iron sulfide (black non-magnetic precipitate, probably Fe(II) monosulfide), chemically synthesized by adding 0.5 ml of a Na_2_S*9H_2_O solution (50 mM) to 10 ml of the medium with ferrihydrite (90 mM of Fe(III) per liter). Glauconite and iron sulfide were added to or synthesized in the medium prior to sterilization.

### Preparation and processing of the samples

*T*. *siderophilus* was inoculated (5% v/v) into the medium with ferrihydrite, glauconite, or iron sulfide and cultivated for 47 hours. Seventy-two aliquots of 2 ml of outgrown liquid cultures with a cell density of 2.0–2.3*10^8^ cells/ml, of which 15–20% of cells had endospores, were dried under the air at an ambient temperature. The average mass of a dried sample was 0.0148 ± 0.002 g for magnetite/siderite, 0.0181 ± 0.003 g for glauconite, and 0.0164 ± 0.002 g for iron sulfide. Each sample contained approximately 4*10^8^ cells (vegetative cells + cells with endospores). Each dried sample was placed into a separate well drilled in the basalt disc. The wells were closed with Teflon stoppers and then sealed with the composite resin cement Estelite ∑ Quick (Tokuyama Dental Corporation, Japan). After the flight the seals were aseptically removed using a Saeshin Forte-200 portable drilling machine equipped with a diamond dental drill (SS White). The complete content of each sample was transferred into 10 ml of liquid anaerobic cultivation medium with peptone and ferrihydrite and incubated at 65°C. The samples from test, orbital, and ground controls were processed in the same way.

### Construction of the artificial meteorite

**Fig 1:**
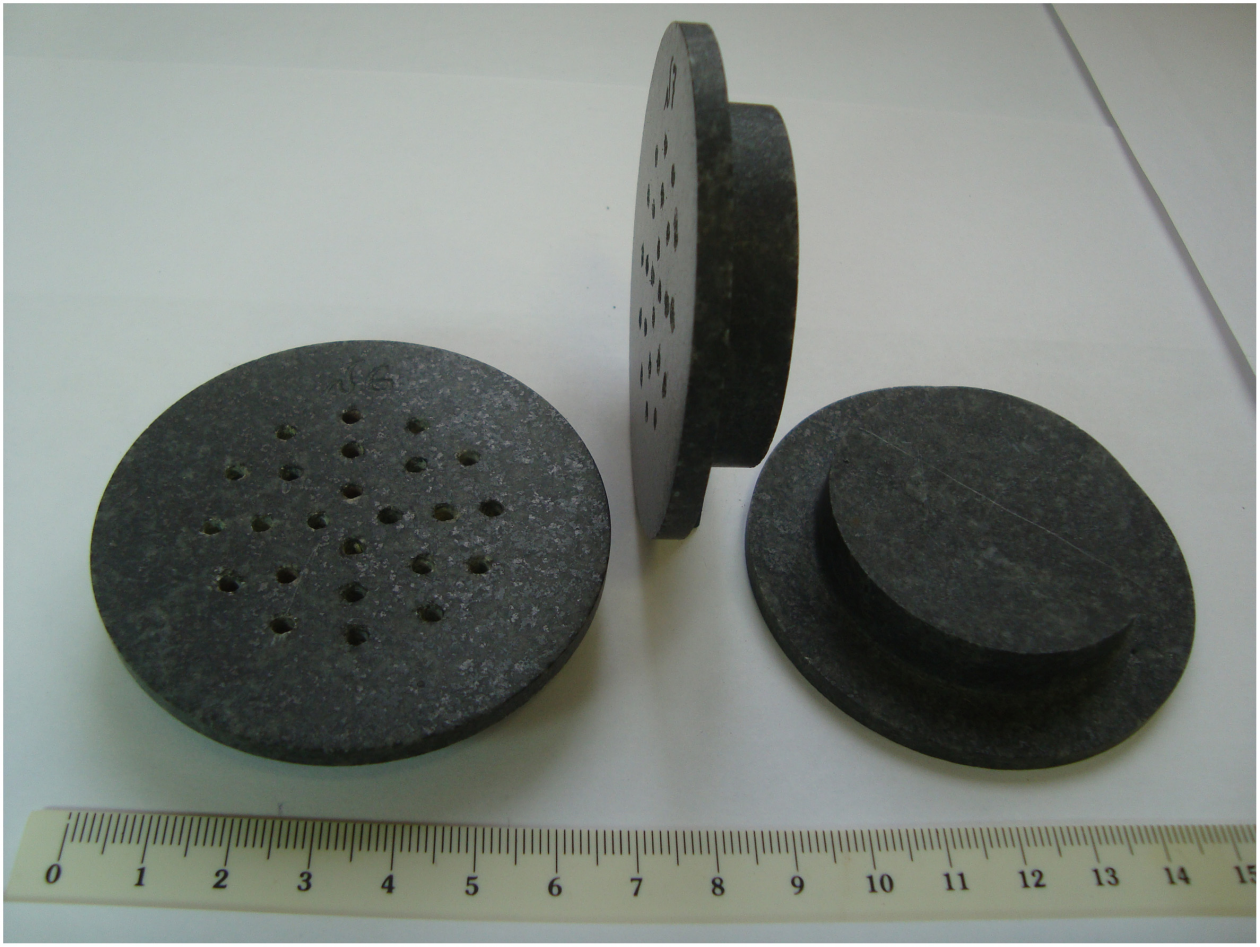
The basalt discs before loading with microbial cultures.

**Fig 2:**
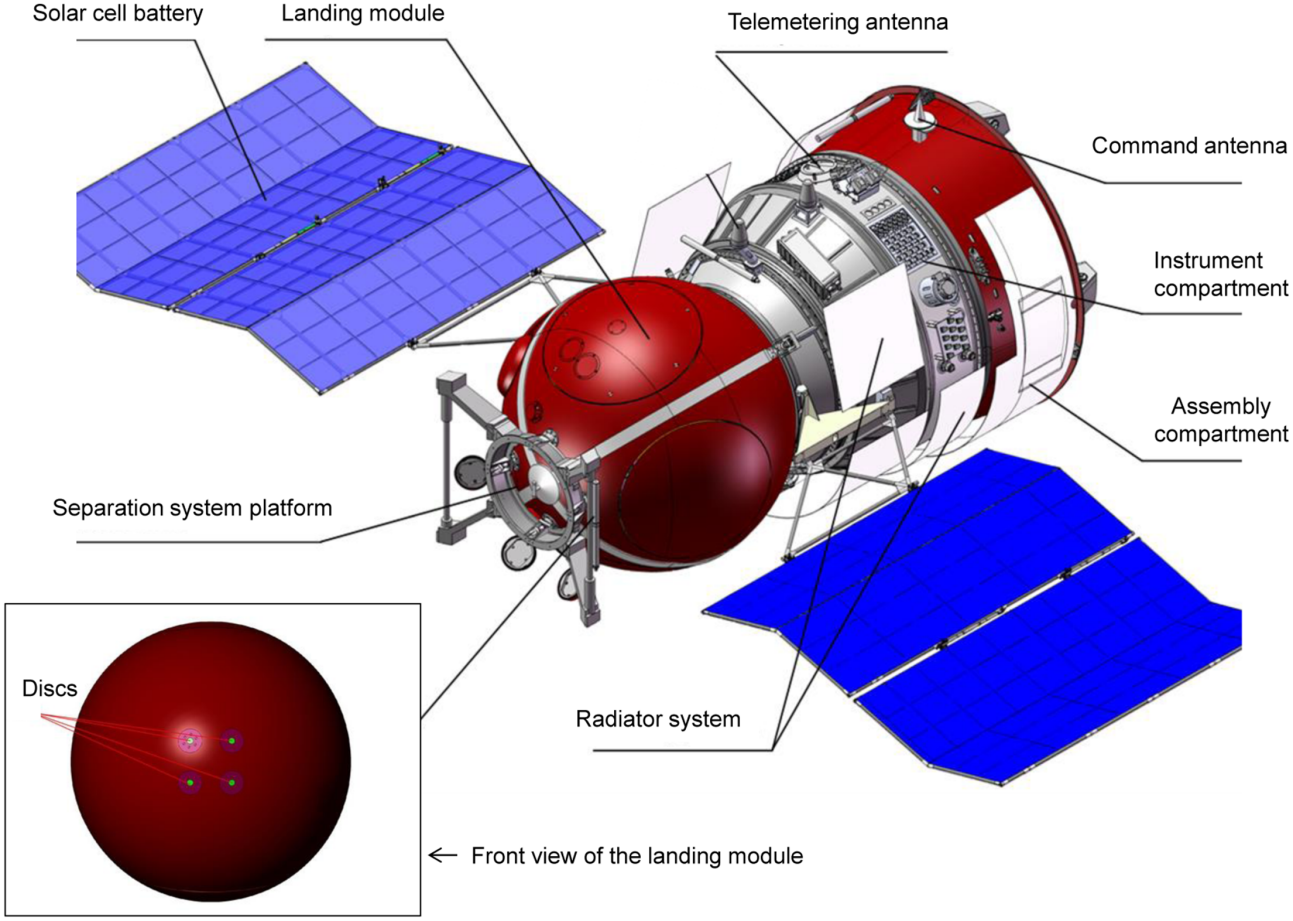
Schematic illustration of FOTON-M4 satellite showing the position of the METEORITE experiment payload. Modified from original drawing supplied by the Russian Federal Space Agency at http://roscosmos.ru/20669/.

Six 1.4-cm-thick basalt (from the mineralogical collection of Lomonosov Moscow State University) discs with a diameter of 7.0 cm were used as a model of the artificial meteorite in this part of the METEORITE experiment (Fig 1). For the placement of the dried microbial cultures, 24 wells, 2 mm in diameter, were drilled on the back side of the disc to a depth of 6 mm. Twelve wells in each disc were filled with dried cultures of *T*. *siderophilus*, while the other 12 wells were filled with cultures of other microorganisms (this will be the subject of a separate report). Prior to inoculation, the discs were wrapped in aluminum foil and sterilized by autoclaving (135°C, 2 h). After loading with microbial cultures, four discs were inserted into the holders (annular discs of phenolic silica, i.e. the same material as was used for the ablative capsule heat shield) and fixed on the exterior of the FOTON-M4 capsule. Two discs were fixed near the stagnation point (test), and two discs were fixed on the reverse side of the landing module (orbital control) (Fig 2). Two other discs were kept in the laboratory at ambient temperature (ground control).

### Description of flight

The FOTON-M4 space vehicle was launched by a Soyuz-2.1a launcher rocket from Baikonur (Kazakhstan) on July 19, 2014. On September 1, after 45 days in Earth orbit (mean orbit: 575 km), the velocity of the capsule was decreased slightly from 7.7 to 7.6 km/s for atmospheric entry. During atmospheric entry, the landing module was travelling almost parallel to the surface of the Earth. The parachute was deployed 24 min later at an altitude of 9 km for landing in a field in the Orenburg oblast, Russia. Because a parachute was used, the landing module experienced a “soft” landing (ca. 40 m/s) rather than a ballistic impact. Thus, only high-speed entry and not the conditions of collision of a meteorite with the Earth’s surface was simulated in the METEORITE experiment.

### Other methods

Before and after the flight test, the control discs were handled as aseptically as possible, using sterile plastic bags and disposable gloves. All manipulation involving microbial cultures, including loading, sealing, removal of the seals, inoculation, and so on, was performed in a pre-sterilized laminar flow cabinet. The growth of bacteria was determined by direct counting of the cells and spores in the aliquot of a liquid culture with a phase contrast microscope (Olympus CX41) and a counting chamber. Reduction of Fe(III) was judged from the formation of a black magnetic material from brown non-magnetic ferrihydrite using a hand magnet. Fe(III) reduction was monitored by measuring the accumulation of Fe(II) over time. Fe(II) was analyzed by adding a 0.5 ml sample from the culture to 5 ml of 0.6 M HCl. After 24 hours of extraction, HCl-soluble Fe(II) was determined with 2,2’-dipyridyl [26]. The DNA was extracted by the method of Marmur [27] and purified using Wizard Maxipreps DNA Purification Resin (Promega, Madison, WI, USA). The 16S rRNA gene was selectively amplified from genomic DNA by PCR using 27F and 1492R forward and reverse primers, respectively [28], as previously described [24]. The 16S rRNA gene was sequenced by means of Big Dye Terminator v. 3.1 (Applied Biosystems, USA), as described in the manufacturer’s instructions, using an ABI PRISM 3730 sequencer (Applied Biosystems, USA). The sequences were assembled and checked for accuracy manually using the alignment editor Bio-Edit v. 5.0.9 [29]. The nearly full-length 16S rRNA gene sequence (1562 nt) was compared with other sequences in GenBank [30] using BLAST [31] to identify the closest relatives. Determination of the number of cultivable vegetative cells/endospores was performed through serial dilutions in a liquid cultivation medium in duplicate for orbital and ground control samples.

## Results

**Fig 3:**
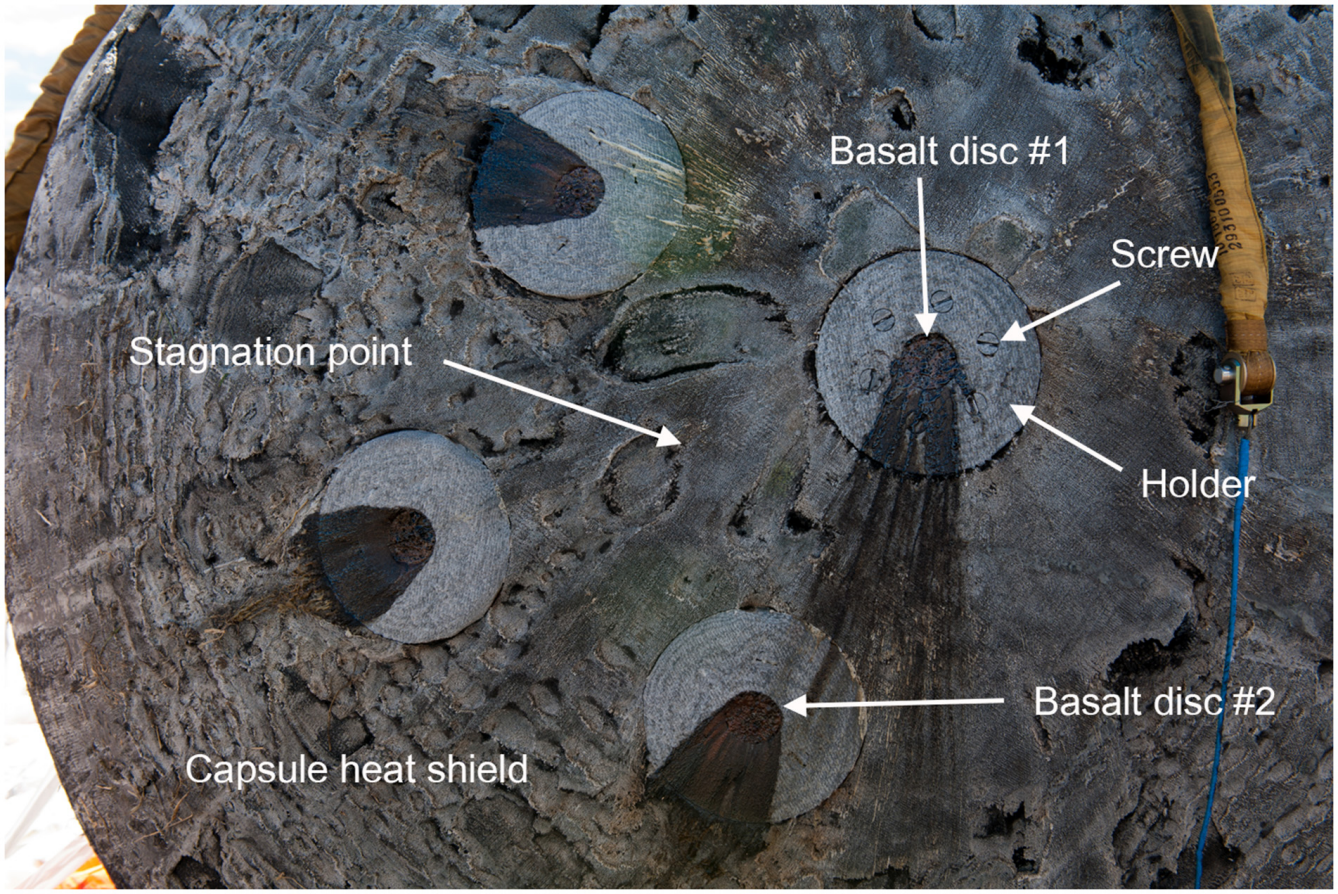
FOTON-M4 landing module after landing, showing the placement of the discs around the stagnation point. Two of four discs fixed near the stagnation point were used in this part of the METEORITE experiment.

In the METEORITE experiment, four basalt discs loaded with dried microbial cultures (Fig 1) were fixed on the exterior of the space capsule of the FOTON-M4 satellite. After 45 days of orbital flight, the landing module was returned to Earth (Fig 3). Discs #1 and #2 were exposed to conditions of orbital flight and hypervelocity entry from space through the atmosphere (test). Discs #3 and #4 were exposed to conditions of orbital flight but were protected from heating during reentry by the radio-controlled lids (orbital control). Two other discs (#5 and #6) were kept in the laboratory at ambient temperature (ground control).

**Fig 4:**
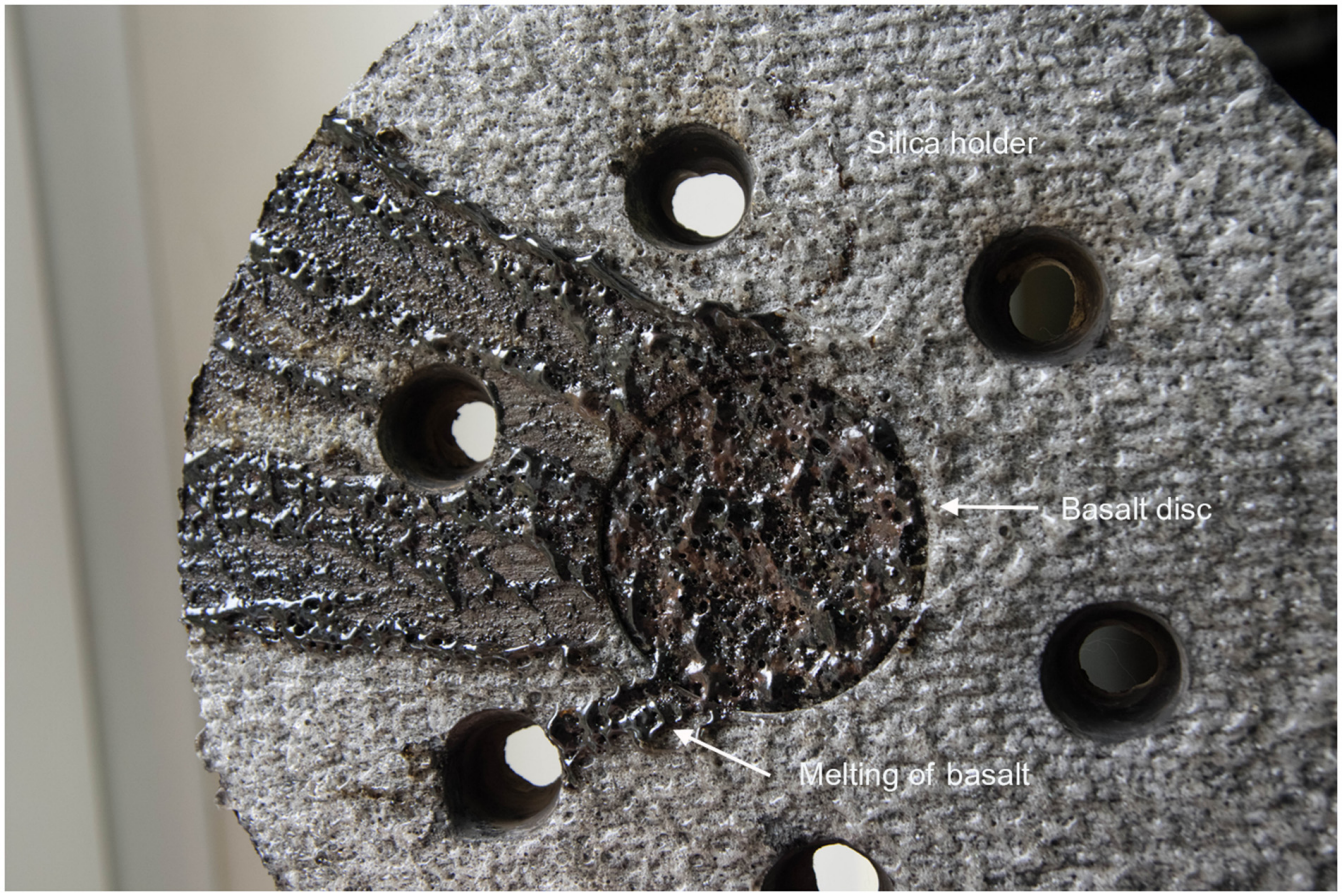
Exterior of the test basalt disc (Disc #1) exposed to atmospheric entry. Note the melting of the basalt.

**Fig 5:**
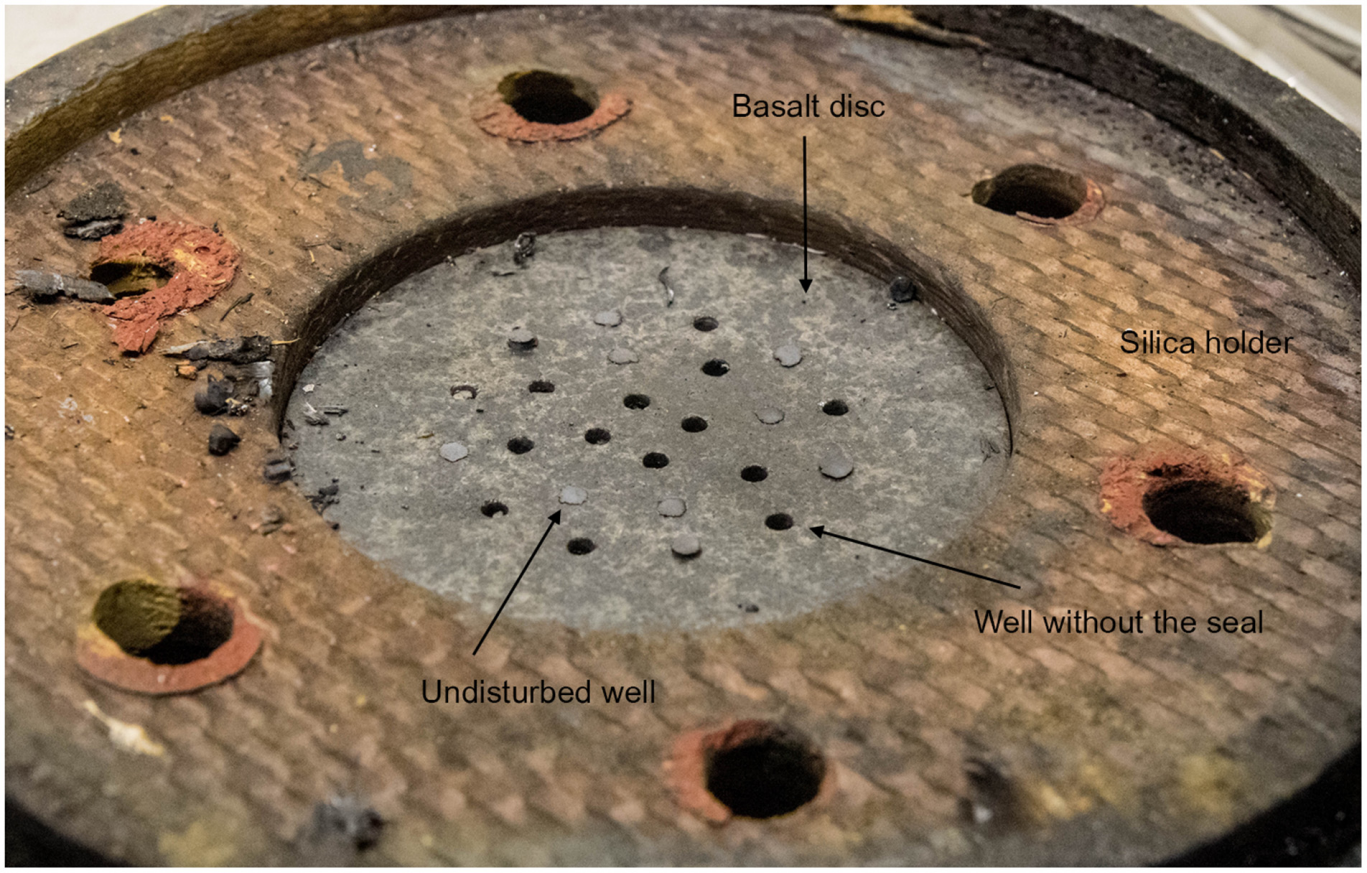
Interior of the test basalt disc (Disc #1) after the flight. The back side of the disc with the undisturbed wells and the wells without the seals is shown.

**Fig 6:**
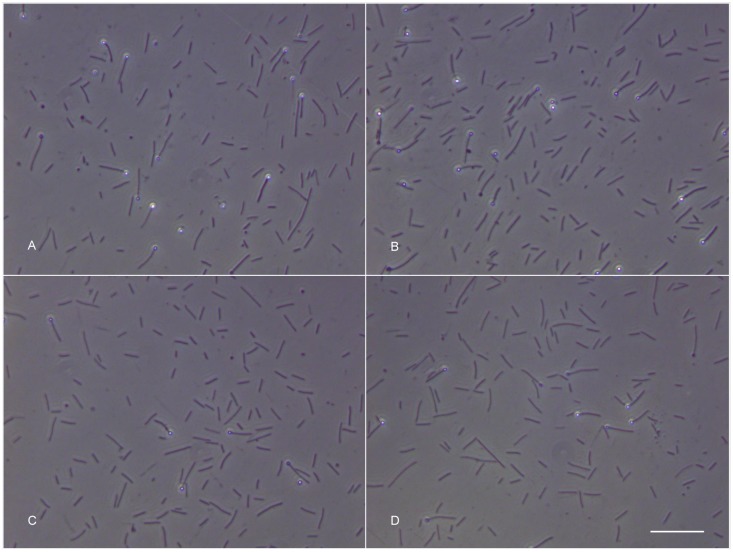
**Morphology of the cells recovered from:** (A) Disc #1 (test), (B) Disc #3 (orbital control), and (C) Disc #5 (ground control). (D) Collection culture of *T*. *siderophilus* SR4^T^. Bar, 10 μm.

**Fig 7:**
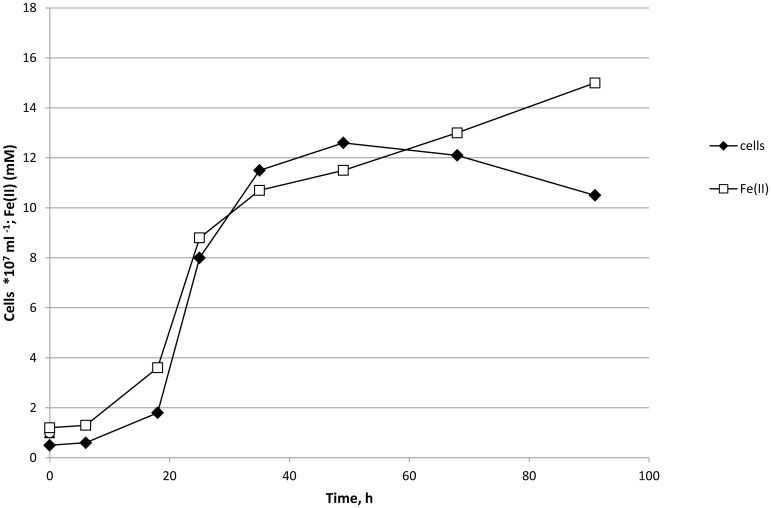
Growth of, and Fe(II) production by *T*. *siderophilus* survived the atmospheric entry. The data presented for the culture that was recovered from the undisturbed well and then subsequently transferred three times [5% (v/v) inoculum] in liquid anaerobic medium with peptone and ferrihydrite.

The temperature during the atmospheric transit was not instrumentally recorded but was high enough to melt the surface of basalt (melting temperature: 1,100–1,250°C) (Fig 4). After the landing, some wells in test discs #1 and #2 had lost their seals; however the dried microbial cultures within the wells were still present (Fig 5). Inoculation of the dried cultures recovered from the wells into a liquid cultivation medium followed by incubation at 65°C for 120 hours resulted in microbial growth and ferrihydrite reduction in 4 of the 24 cultures (Table 1). Further incubation of 20 growth-negative cultures during the next three months did not cause growth and Fe(III) reduction. In orbital (24 wells) and ground (24 wells) control discs, all wells retained their seals, and microbial growth and Fe(III) reduction were observed after inoculation of dried cultures recovered from 48 wells (100% survival). In growth-positive cultures the concentration of the cells after 120 hours was 8–10*10^7^ cells/ml, with 15–20% of the cells containing endospores. The morphology of the cells obtained from the test, orbital control, and ground control was consistent with the original description of *T*. *siderophilus* [23] (Fig 6). The culture recovered from the undisturbed well of disc #1 was capable of sustainable growth and Fe(III) reduction (Fig 7). After three subsequent 5% (v/v) transfers, specific rates of growth and Fe(II) production of the culture survived the atmospheric entry and the cultures recovered from orbital and ground controls were equal within the range of statistical error (Table 2). A nucleotide sequence of the 16S rRNA gene (1562 b.p.) of the culture that survived the atmospheric entry was 100% identical to the type strain of *T*. *siderophilus* (GenBank Accession NZ_CM001486). The sequence has been deposited in GenBank under accession number KR736354. Thus the morphological and physiological characteristics as well as a nucleotide sequence of the 16S rRNA gene of the culture survived the atmospheric entry were identical to the type strain of *T*. *siderophilus* SR4^T^ loaded into the artificial meteorite before the flight.

**Table 1:** Revival of *T*. *siderophilus* from dried cultures exposed to hypervelocity atmospheric entry.

Wells	Mineral		
	Magnetite/siderite (3 wells per disc)	Glauconite (6 wells per disc)	Iron sulfide (3 wells per disc)
Disc #1			
Total number of undisturbed wells	3	3	1
Number of undisturbed wells from which growth of the microorganism was obtained	0	1	0
Total number of wells without a seal	0	3	2
Number of wells without a seal from which growth of the microorganism was obtained	0	1	1
Disc #2			
Total number of undisturbed wells	2	2	1
Number of undisturbed wells from which growth of the microorganism was obtained	0	0	0
Total number of wells without a seal	1	4	1
Number of wells without a seal from which growth of the microorganism was obtained	0	1	0

The cultures grown in the medium with different iron minerals were loaded into the wells drilled in the basalt discs.

**Table 2:** Specific rates of growth and Fe(II) production (mean ± standard deviation of three independent experiments) in cultures of *T*. *siderophilus*.

Culture	Specific growth rate (hour^-1^)	Specific rate of Fe(II) production (hour^-1^)
Test	0.247 ± 0.041	0.146 ± 0.032
Orbital control	0.223 ± 0.037	0.126 ± 0.028
Ground control	0.265 ± 0.049	0.159 ± 0.034

Attempts to estimate the numbers of microbial cells/spores that survived the entry using the serial dilution technique were unsuccessful; no growth was detected in the four undisturbed wells (one with magnetite/siderite, two with glauconite, and one with iron sulfide) selected for this task (Table 3). In the orbital control, the number of cultivable vegetative cells/endospores decreased 10–1,000 times in comparison with the ground control.

**Table 3:** Number of cultivable vegetative cells/endospores in the dried cultures of *T*. *siderophilus* after atmospheric entry (test) and in the orbital and ground controls.

Mineral	Number of cultivable cells/spores		
	Test	Orbital control	Ground control
Magnetite/siderite	0	10^1^	10^4^
Glauconite	0 (10^1^)*	10^3^	10^4^
Iron sulfide	0 (10^1^)*	10^2^	10^4^

* Growth was obtained in initial incubation from another well, which was conducted in parallel but without determination of the number of cells/spores.

The recovery of dried microbial cultures from test discs #1 and #2 was performed before the processing of orbital and ground control discs to avoid cross-contamination with *T*. *siderophilus*. In order to estimate possible microbial contamination of the laboratory environment, an open beaker with 50 ml of distilled water and a handful of glauconite was placed just after recovery of the microbial cultures from the ground control discs into the same non-sterilized laminar flow cabinet and left for 8 hours. Then the aliquots of distilled water and glauconite were inoculated (2% w/v) into a liquid anaerobic cultivation medium with peptone and ferrihydrite in the same manner as was done for the test samples and incubated at 65°C. Neither cell growth nor Fe(III) reduction was observed in these incubations over a period of three months.

## Discussion

The results of our study demonstrate that the spore-forming thermophilic anaerobic bacterium *Thermoanaerobacter siderophilus* survived entry into the Earth’s atmosphere within an artificial meteorite. This experimental evidence supports the possibility of interplanetary transport of lifeforms and makes the lithopanspermia hypothesis more realistic. Finds of several dozen Martian meteorites on Earth together with numerical computations indicate that the interchange of bioactive material between Mars and Earth is highly probable [1, 2]. Billions of Martian meteorites have fallen on Earth since the dawn of our planetary system; about 0.5% of them should have travelled in space for not more than 1 million years; besides that many Martian meteoroids make the journey in less than a century, and a few potentially make it in less than a decade [1]. At travel times of 10^4^ to 10^5^ years, a regolith thickness of a few tens of centimeters should be sufficient for effective protection of microbial spores from solar UV radiation, X-rays, and galactic cosmic rays [1]. Our data show that the Earth’s atmosphere is also not an impassible barrier for microbial spores residing within rocky meteorites. The velocity of the FOTON capsule during the entry (7.6 km/sec) was somewhat lower than the velocity of medium-sized natural meteorites (10–20 km/sec), although it was still high enough to melt basalt, indicating that the temperature of the surface was more than 1,100°C. The formation of a fusion crust of melted rock caused by heating and ablation is typical for natural meteorites [32]. The temperature distribution under the crust surface in our experiments was not recorded. It is difficult to estimate the actual temperatures within natural meteorites based on theoretical calculations. Paleomagnetic studies of the Martian meteorite ALH84001 demonstrated that only 3 mm below the surface of its fusion crust, the interior had never been heated to a temperature of more than about 40°C during its entire history of ejection from Mars, interplanetary transit to Earth, and passage through Earth’s atmosphere [33]. However, many meteorites break up into fragments during entry, so the putative microbial population initially residing deep within a rock would become exposed to high temperatures in the newly formed near-surface sites. Thus, in the case of a meteorite splitting, heat-resistant microorganisms or spores would have a higher chance of surviving. Hypothetically, a thickness of a few centimeters seems to be reliable for complete protection of the microorganisms from extensive heating. However, so far all experimental attempts to demonstrate the survival of mesophilic microorganisms and spores located within artificial meteorites at a depth of 1–2 cm that entered the atmosphere at velocities of more than 7.5 km/sec have been unsuccessful [16–19]. It was suggested that the living organisms probably need to be protected by at least 5 cm of rock in order to be shielded from the intense heat of entry [18]. Our data prove that a layer of basalt with a thickness of 1.4 cm protects spore-forming thermophilic microorganisms from the heating generated by atmospheric entry of a meteorite at a velocity of 7.6 km/sec.

In this study, we did not separate the vegetative cells and endospores of *T*. *siderophilus* before the placement of the dried cultures into the artificial meteorite. It is most likely that only the endospores survived the harsh conditions of the entry and that all vegetative cells were inactivated by heat. The number of surviving microbial cultures was rather low. *T*. *siderophilus* was recovered from one undisturbed well and from three wells without their seals out of the total of 24 wells loaded with this bacterium and situated on two distinct test discs (Table 1). The presence of viable spores in wells without their seals is not a result of contamination, since no viable spores were recovered from other wells located on the same test discs, from sterile wells in the ground control samples, or from contamination control incubations. The observed rate of survival, 25% for Disc #1 and 8% for Disc #2, probably reflects non-uniform heating and temperature distribution in the discs during the entry caused by the slightly different inclination of the discs relative to the trajectory of the entry. Unfortunately, we failed to estimate the number of surviving spores in each well due to the limited number of wells and the low recovery rate. Prior to the flight, each separate well was loaded with a dried culture of *T*. *siderophilus* that contained approximately 4*10^8^ cells (vegetative cells + cells with endospores) and about 6*10^7^ endospores (free or within the sporangia) absorbed on 15–20 mg of iron-containing mineral precipitate. The obtained results show that this density of cells/spores is sufficient for the survival of at least one spore. In orbital and ground controls, the number of viable spores decreased by 10^4^–10^6^ times and 10^3^ times respectively. This could be explained by the influence of outer space parameters (radiation, microgravity, etc.) on the orbital samples and by the adverse effect of oxygen on the survival of spores of anaerobic microorganisms.

In our experiment, iron-containing minerals were used as excipients in dry microbial cultures. Different iron minerals are common constituents of endolithic environments; furthermore, many of these compounds could be effective adsorbents due to their large specific surface area. Fine-grained magnetite (Fe_3_O_4_) and siderite (FeCO_3_) are products of the bio-reduction of ferrihydrite by numerous dissimilatory Fe(III)-reducing microorganisms, including *T*. *siderophilus* [34]. Fine-grained single-domain magnetite was found in the Martian meteorite ALH84001 [35]. Glauconite is iron potassium phyllosilicate of the mica group that is characteristic of marine sediments. Iron sulfides are found in meteorites originating from Mars and the Moon [36]; troilite (FeS) is the most common sulfide mineral in lunar rocks [37]. In our study, the survival of *T*. *siderophilus* was documented in three cultures with glauconite and in one culture with iron sulfide, but no viable cells were recovered from cultures with magnetite/siderite. The number of surviving cultures is too low and does not allow us to conclude which mineral is the best excipient. The survival of spores in certain wells could be connected not with the particular mineral but with other factors, most probably with the temperature distribution in the disc. Theoretically, the structure of the mineral could determine the level of absorption of spores; moreover, differences in the thermal conductivity of various minerals could influence the degree of protection of spores during rapid and intensive heating. In any case, the results of this study demonstrate that bacterial endospores absorbed on iron silicates and iron sulfides inside an artificial stony meteorite can withstand the conditions of hypervelocity atmospheric entry.

In conclusion, our results show for the first time that bacterial spores can survive within an artificial meteorite after entry from space orbit through the Earth’s atmosphere at a velocity that closely approaches the velocities of natural meteoroids. This is the first successful experiment on the survival of lifeforms performed on the landing modules of space vehicles. The characteristics of the artificial meteorite (material, thickness, and excipients for microorganism preservation) and the living object (spore-forming thermophilic anaerobic bacterium) applied in this study can serve as positive controls in further experiments on the testing of different organisms and conditions of interplanetary transport.
